# NMR Study of Water-Soluble Carotenoid Crocin: Formation of Mixed Micelles, Interaction with Lipid Membrane and Antioxidant Activity

**DOI:** 10.3390/ijms25063194

**Published:** 2024-03-11

**Authors:** Wenjing Su, Anna V. Mastova, Maya A. Ul’yanova, Polina A. Kononova, Olga Yu. Selyutina, Veronika I. Evseenko, Elizaveta S. Meteleva, Alexander V. Dushkin, Weike Su, Nikolay E. Polyakov

**Affiliations:** 1 Collaborative Innovation Center of Yangtze River Delta Region Green Pharmaceuticals, Zhejiang University of Technology, Hangzhou 310023, China; 1112123010@zjut.edu.cn (W.S.); dushkin@solid.nsc.ru (A.V.D.); pharmlab@zjut.edu.cn (W.S.); 2 Institute of Chemical Kinetics and Combustion, 630090 Novosibirsk, Russia; mastova-anna99@yandex.ru (A.V.M.); ulyanova@kinetics.nsc.ru (M.A.U.); kononova_polina@bk.ru (P.A.K.); gluschenko@gmail.com (O.Y.S.); 3 Institute of Solid State Chemistry and Mechanochemistry, 630128 Novosibirsk, Russia; evseenkov@inbox.ru (V.I.E.); meteleva1182@gmail.com (E.S.M.)

**Keywords:** carotenoids, crocin, glycyrrhizin, mixed micelles, lipid peroxidation, vitamin C, NMR, NOESY, molecular dynamics simulation

## Abstract

Crocin is a unique water-soluble carotenoid found in crocus and gardenia flowers. Crocin has been shown to have a variety of pharmacological activities, such as antioxidant, anti-cancer, memory improvement, antidepressant, anti-ischemia, blood pressure lowering and aphrodisiac, gene protection and detoxification activities. Due to their amphiphilicity, crocin molecules form concentration-dependent self-associates (micelles) in a water solution. In the present study, using various NMR techniques (T_2_ relaxation and selective gradient NOESY), we have demonstrated that crocin forms mixed micelles with water-soluble drug delivery system glycyrrhizin and linoleic acid molecules. Note, that the spin–spin T_2_ relaxation time and NOESY spectroscopy are very sensitive to intermolecular interactions and molecular diffusion mobility. The second purpose of this work was the elucidation of the interaction of crocin with a model lipid membrane using NMR techniques and a molecular dynamics simulation and its effects on lipid oxidation. It was shown that the crocin molecule is located near the surface of the lipid bilayer and effectively protects lipids from oxidation by peroxyl radicals. The role of glycyrrhizin and vitamin C in metal-induced lipid oxidation was also elucidated. The results of this study may be useful for expanding the field of application of crocin in medicine and in the food industry.

## 1. Introduction

**Figure 1:**
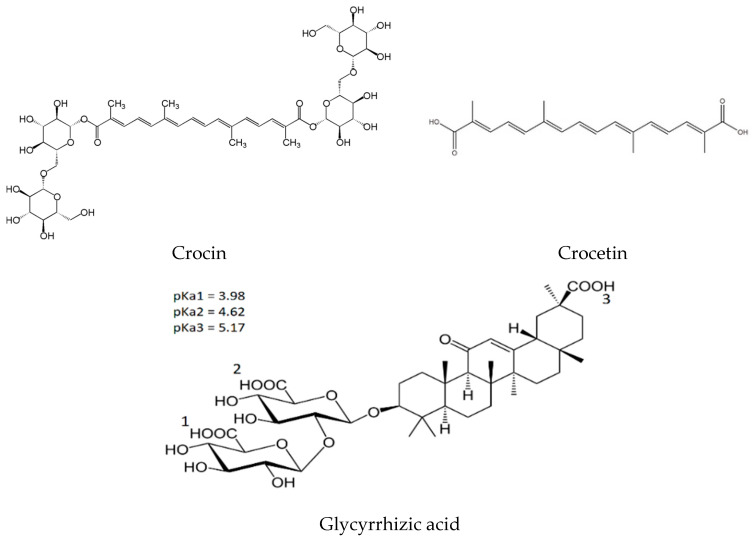
Structure of crocin, crocetin, and glycyrrhizic acid.

Crocin (C_44_H_64_O_24_, Figure 1) is a water-soluble carotenoid extracted from the stigmas of *Crocus sativus* L. flowers, which are mainly grown in Iran, Greece, India, Italy, and other countries. It is a glycosyl ester derivative of the C20-dibasic acid crocetin (8,8’-dicarotenoic acid) (Figure 1). Crocin has pharmacological activities such as antioxidant, anti-inflammatory, anti-cancer, and anti-atherosclerosis activities and others [1,2,3,4,5,6]. The advantage of crocin over other carotenoids is its good water solubility, safety, and excellent physiological activity; thus, it is also used as a dye and food additive [2,7]. As one can see from Figure 1, crocin is an amphiphilic compound. It contains both a central hydrophobic part (unsaturated polyene chain) and side hydrophilic parts (glycosyl fragments). It is well known that such amphiphilic compounds are prone to self-association in aqueous solutions, with the formation of dimers, micelles, liposomes, or gel nanoparticles [8,9,10,11,12]. As for crocin itself, several studies have been performed to study its aggregation ability in aqueous solutions as well as its interaction with other amphiphilic compounds [13,14,15]. In particular, Z. Khan and coauthors studied the self-aggregation behavior of crocin and mixed micelle formation with sodium dodecyl sulfate (SDS) and cetyltrimethylammonium bromide (CTAB) using dynamic light scattering (DLS) and transmission electron microscopic (TEM) techniques. It was concluded that pure crocin forms vesicles in a water solution, whereas vesicle-to-micelle transformation was observed in the presence of CTAB [13]. Various physicochemical techniques, such as UV–visible absorption spectroscopy, dynamic light scattering, transmission electron microscopy, energy-dispersive X-ray spectroscopy, and selected area electron diffraction patterns, have been also applied by Z. Zaheer and coauthors to study aqueous solutions of crocin and its self-aggregates [14]. The critical micellar concentration of crocin was determined as 0.2 mM. Using TEM images and DLS results, the shape, size, and size distribution of crocin aggregates have been characterized. The interaction of crocin with bovine serum albumin (BSA) was studied using various techniques such as surface tensiometry, UV–visible spectrophotometry, and fluorescence spectroscopy [15].

However, similar to most other carotenoids, the application of crocin in medicine and the food industry is restricted by its instability when exposed to heat, oxygen, light, acidic environments, and metal ions [16,17,18]. For example, the optical properties of crocin change, that is, the absorbance at short wavelengths increases, resulting in a decrease in the color rendering ability of crocin [19,20]. A high temperature will break the glycosidic bond of crocin, and the glucose molecules will be lost, thereby reducing or even eliminating its physiological activity [20].

To increase the stability and bioavailability of lipophilic carotenoids like β-carotene, lutein, astaxanthin, and others, the formation of inclusion complexes with various supramolecular drug delivery systems (DDS) is used as an effective approach [21,22,23,24]. It has previously been demonstrated that this approach allows not only an increase in the stability and bioavailability of carotenoids but also corrects their antioxidant and photophysical properties [21,25]. In particular, the incorporation of some carotenoids into polymer nanoparticles allows an increase in their photostability by 5–10 times. It was shown that the main mechanism of increasing the photostability of carotenoids in aqueous solution is the isolation of carotenoids from water molecules, which play the role of the proton acceptor from the carotenoid radical cations formed under irradiation. The neutral carotenoid radicals formed in this reaction are very unstable and can rapidly transform into oxidized products. A similar protection mechanism is implemented against transition metal ions, namely iron and copper, which effectively oxidize carotenoids via an electron transfer mechanism [18]. Apanasenko and coauthors have demonstrated that the incorporation of the carotenoids zeaxanthin and lutein into the micelles of the disodium salt of glycyrrhizic acid decreases the oxidation rate of these carotenoids by ferric ions by more than 10 times [25].

However, it should be noted that for most drug delivery systems, the driving force of inclusion complex formation is hydrophobic interaction. Lipophilic molecules, like carotenoids, can easily penetrate the hydrophobic interior of DDS (cyclodextrins, micelles, lipid bilayer, and so on). Therefore, such an approach might be not suitable for the water-soluble carotenoid crocin. On the other hand, as mentioned above, due to their amphiphilicity, crocin molecules can form concentration-dependent self-associates (micelles or vesicles) in a water solution [13,14,15]. Also, crocin molecules are able to be incorporated into the micelles formed by the surfactants SDS and CTAB. We have suggested that crocin is also able to form mixed micelles with other amphiphilic molecules used as the DDS, for example, with lipid molecules and saponins (see reviews [26,27,28,29] for the application of lipid nanoparticles and saponins as the drug delivery systems). To prove such a possibility, in the present study, we have tried to use saponin glycyrrhizin and lipid molecules to prepare mixed micelles with crocin. Note, that only a few examples of DDS for crocin molecules have been reported so far [30,31].

Glycyrrhizin or glycyrrhizic acid (GA, Figure 1) is the main bioactive component of licorice root (*Glycyrrhiza glabra* and *G. uralensis*). Licorice is one of the most frequently used plants in traditional Eastern medicine [32,33,34,35]. GA has been established to be safe for food and medical applications [36,37,38] and has a wide range of biological activities such as anti-inflammation, anti-viral, and anticancer activity (see review [39]). In previous studies, it was demonstrated by various physicochemical methods that the amphiphilic nature of GA allows it to form micelles and gel nanoparticles in aqueous solutions as well as inclusion complexes with drug molecules, thereby enhancing their stability, permeability, and bioavailability [40,41]. The formation of inclusion complexes of GA has been detected not only in aqueous solutions but also in other organic solvents DMSO and acetonitrile [42,43].

In the present study, we have applied various NMR techniques (T_2_ relaxation and selective gradient NOESY) to answer the question of whether crocin forms mixed micelles with saponin glycyrrhizin and linoleic acid molecules. Note, that the spin–spin T_2_ relaxation time and NOESY spectroscopy are very sensitive to intermolecular interactions and molecular diffusion mobility. The second purpose of this work was the elucidation of crocin interaction with a model lipid membrane using NMR techniques and a molecular dynamics simulation and the effect of crocin on lipid oxidation. The role of glycyrrhizin and vitamin C in transition metal-induced lipid oxidation was also elucidated.

## 2. Results and Discussion

### 2.1. NMR Study of Crocin/GA Mixed Micelles in Water Solution

**Figure 2:**
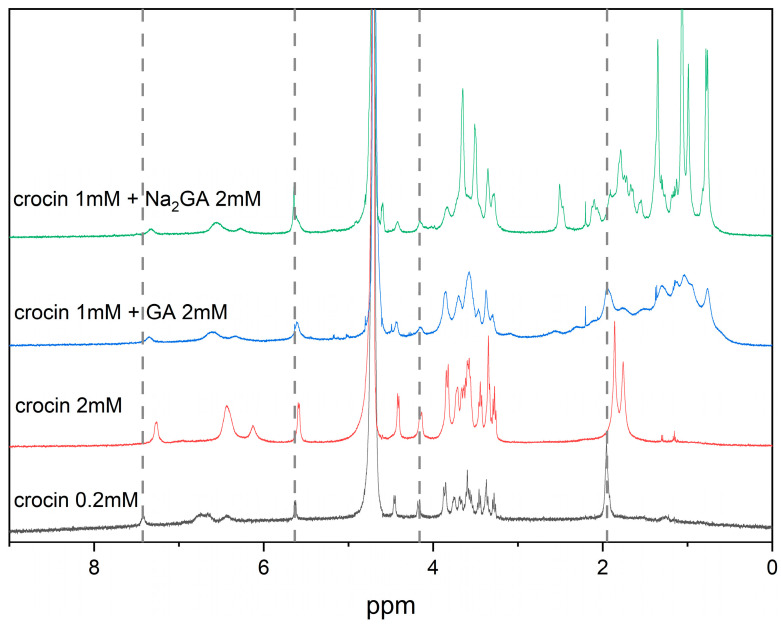
^1^H NMR spectra of crocin in PBS (pH = 7.1): 0.2 mM, 2 mM, 1 mM + 2 mM GA, 1 mM + 2 mM Na_2_GA.

Various NMR techniques, including NOESY spectroscopy and NMR relaxation study, are very sensitive to intermolecular interactions. That is why these techniques are widely used to study self-association processes and inclusion complex formation [12,25,44,45]. Earlier, it was shown that due to their amphiphilicity, crocin molecules are able to form self-aggregates in a water solution [13,14,15]. The critical micellar concentration (CMC) of crocin was estimated at 0.2 mM. Figure 2 shows the ^1^H NMR spectra of water crocin solutions at different concentrations (0.2 mM and 2 mM). One can see the changes in the chemical shifts of the methyl protons (around 2 ppm) and =CH– protons (5.5–7.5 ppm) but not of the sugar protons (3–4.5 ppm) due to the self-association of crocin into micelles in the water solution. The NMR relaxation study showed a decrease in the T_2_ proton relaxation time from 130 ± 50 milliseconds to 20 ± 5 milliseconds for the methyl protons and from 155 ± 25 to 80 ± 10 milliseconds for the sugar protons when the concentration of crocin was increased from 0.2 to 2 mM. The reason for this effect is that during the formation of aggregates, the diffusion and rotational mobility of the crocin molecules slow down, and the relaxation time of their protons decreases significantly. This also shows that the crocin behavior of self-assembling into micelles in aqueous solution is concentration-dependent.

**Figure 3:**
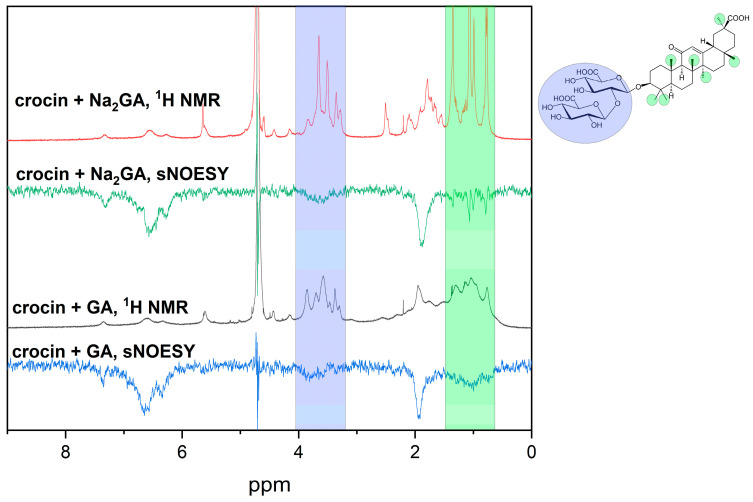
Selective NOESY experiments with crocin in PBS (pH = 7.1). Bottom: 1 mM crocin + 2 mM GA (mixing time = 0.1 s); Top: 1 mM crocin + 2 mM Na_2_GA (mixing time = 0.7 s). Crocin =CH– protons are saturated.

Figure 2 also shows a shift and broadening of the NMR lines of crocin in the presence of 2 mM of GA and its disodium salt Na_2_GA in a buffered water solution. Taking into account the amphiphilicity of the GA molecule and its ability to self-associate, we have suggested that the changes in the NMR spectrum of crocin in the presence of GA and Na_2_GA are due to mixed micelle formation. To prove this hypothesis, we have applied T_2_ relaxation and selective NOESY (sNOESY) techniques, which are extremely sensitive to the diffusional mobility of molecules and intermolecular interactions, as mentioned above. Changes in the T_2_ relaxation time are often considered evidence of inclusion complex formation as well as self-association processes occurring in aqueous solutions [12,44,45]. We expect that when the mixed micelles are formed, their diffusion and rotational mobility will change. From the sNOESY spectra, we expect to detect crocin interaction with the micelles of GA and Na_2_GA. Indeed, the selective gradient NOESY spectra shown in Figure 3 demonstrate the presence of cross-peaks between crocin and the GA protons. The =CH– protons of crocin (6–7.5 ppm) were saturated, and cross-peaks at the position of sugar (3–4 ppm) and methyl GA protons (0.5–1.5 ppm) were detected.

The T_2_ relaxation study showed that the relaxation time of =CH– crocin protons in aqueous solutions (1 mM of crocin) decreases from 18 ± 2 ms to 11 ± 2 ms in the presence of 2 mM GA or Na_2_GA. The significant decrease in T_2_ relaxation time in the presence of GA and Na_2_GA is direct evidence of mixed micelle formation. This NMR relaxation experiment confirmed the conclusions drawn from the results of the sNOESY experiment.

### 2.2. NMR and MD Study of Crocin Interaction with LA Micelles and DMPC/DHPC Bicelles

To shed light on the mechanism of crocin bioactivity, including antioxidant activity, it is important to understand its ability to form mixed micelles or vesicles with lipid molecules as well as the crocin location in the lipid membrane. To answer these questions, in the present study, we have applied a ^1^H-NMR selective gradient NOESY technique and a molecular dynamics simulation. Note, that the intensities of the cross-peaks in the NOESY spectrum are extremely sensitive to the distance between interacting protons. We observed the appearance of a cross-peak in the NOESY spectrum if the distance between the nuclei was less than 0.5 nm. Thus, the arrangement of crocin molecules in the lipid micelle or membrane could be determined.

**Figure 4:**
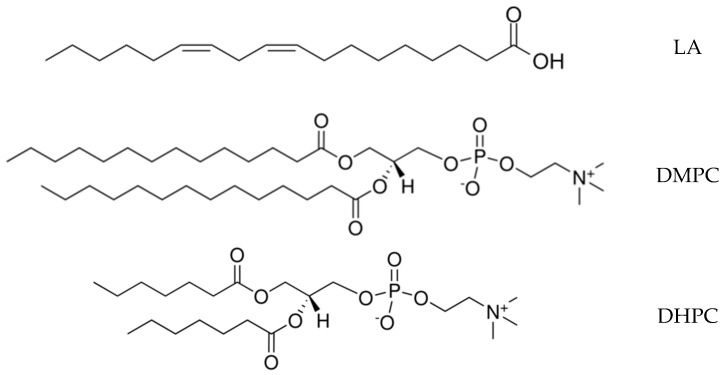
The structures of linoleic acid (LA) and phospholipids DMPC and DHPC used in the present study.

In the present work, we used linoleic acid (LA) to study the mixed micelles of crocin with lipids and as the model for lipid peroxidation and the bicelles DMPC/DHPC to study the crocin interaction with lipid membranes (Figure 4). 

**Figure 5:**
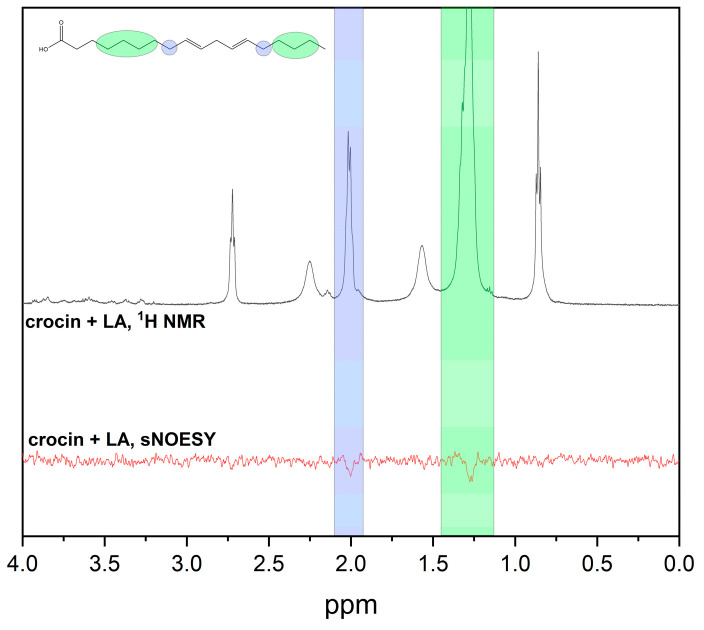
^1^H NMR and selective gradient NOESY spectra of 0.2 mM crocin and micelles of linoleic acid in PBS solution (mixing time = 0.7 s). Crocin =CH– protons are saturated. The presence of cross-peaks indicates penetration of crocin into LA micelles.

Figure 5 shows the presence of a cross-peak in the sNOESY spectrum between the crocin =CH– protons (saturated protons) and the -CH_2_– protons of LA, which indicate their location in the same self-associate. In the next paragraph (Section 2.3), these mixed micelles will be used as a model to study the influence of crocin on metal-induced lipid peroxidation. We assume that the configuration of crocin in mixed micelles with LA is similar to that previously proposed for the crocin molecule included in the SDS and STAB micelles [13]. In that study, the authors suggested that the presence of CTAB significantly changed the aggregation phenomenon of crocin due to the complete solubilization and incorporation of crocin into the CTAB and led to the formation of mixed micelles having a wicket-like conformation.

In the present paper, we deal with mixed micelles, which are large enough and can contain different ratios of molecules in each micelle. So, it is hard to speculate on the exact structure of the self-associates. In the case of linoleic acid micelles, we can assume that the crocin molecule bends in such a way that the sugar fragments are located outside the micelle, and the hydrophobic part is inside. This structure can be inferred from the NOESY data since we observed cross-peaks between the –CH= protons of crocin and the CH_2_ protons of linoleic acid, but we did not observe cross-peaks between the –CH= protons of crocin and the terminal CH_3_ protons of linoleic acid, which would be expected if the crocin molecule was unfolded and pierced the linoleic acid micelle.

**Figure 6:**
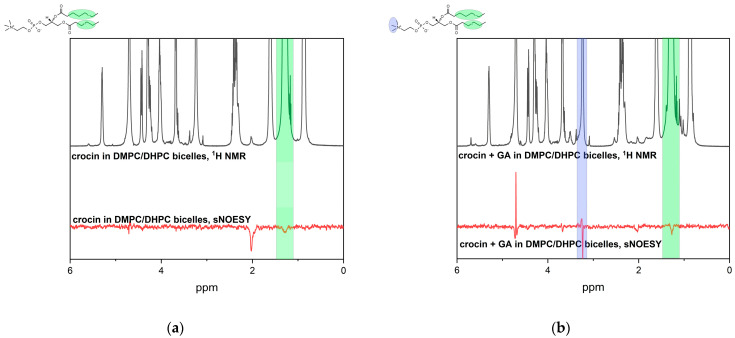
^1^H NMR and selective gradient NOESY spectra of (**a**) 0.5 mM crocin and 24 mM DMPC/DHPC in PBS (mixing time = 0.4 s), (**b**) 0.5 mM crocin + 2 mM GA and 24 mM DMPC/DHPC in PBS (mixing time = 0.4 s). Crocin =CH– protons are saturated. The presence of cross-peaks indicates location of crocin inside lipid bilayer.

Similar effects have been observed for crocin interaction with the model lipid bilayer—bicelles DMPC/DHPC in PBS buffer solution. The presence of cross peaks with the –CH_2_– protons of lipids indicates the location of the hydrophobic chain of crocin inside the lipid bilayer (Figure 6a). The cross-peak at ~2 ppm corresponds to an intramolecular interaction with crocin methyl protons, which are spatially close to saturated crocin =CH– protons. But we do not observe cross-peaks between the CH protons of crocin and the terminal CH_3_ protons of the phospholipid, which would be expected if the crocin molecule penetrated the center of the lipid bilayer. 

When glycyrrhizic acid was added to the system, the additional cross-peak between the crocin =CH– protons and the lipid N^+^(CH_3_)_3_ protons appeared, while the cross-peak between the crocin =CH– protons and the lipid CH_2_-protons remained (Figure 6b). This means that in the presence of GA, crocin is slightly shifted to the bilayer surface but stays immersed in the hydrophobic part of the bilayer. We suppose that the mobility of the CH-chain of crocin increases in the presence of GA, which leads to a freer movement of the investigated protons closer to the surface and back deeper into the bilayer. However, from the experimental data, we can only say unambiguously that some fraction of –CH= protons appears closer to the bilayer surface, which leads to the appearance of cross-peaks between the –CH= protons of crocin and the N^+^(CH_3_)_3_ protons of lipids. The influence of GA on phospholipid membranes has been studied in detail in our earlier works (see review [29] and refs therein).

**Figure 7:**
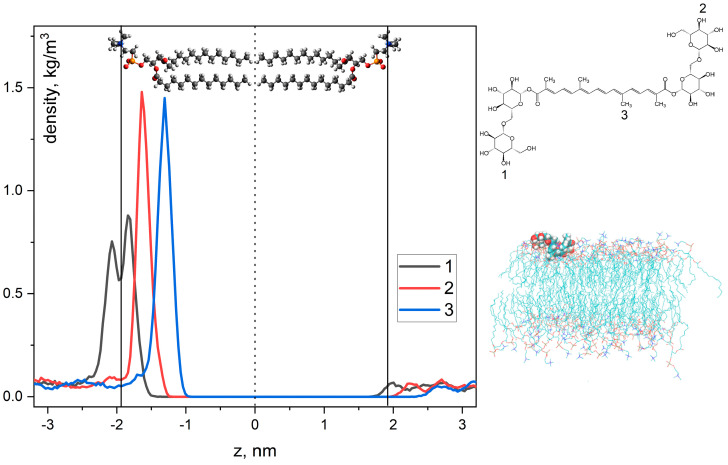
Density profiles of the selected O and C atoms of crocin (**left**). Atoms 1 and 2 belong to OH groups of terminal sugar fragments, and atom 3 belongs to the methyl group in the center of the polyene chain. Vertical lines correspond to the centers of density profiles of DMPC N-atoms. Snapshot of MD trajectory of crocin in box with DMPC bilayer (**right**). Water molecules are not shown. Two production runs with 500 ns duration were performed in MD simulation, and density was calculated by averaging both trajectories.

This experimental result, namely the location of the hydrophobic chain of crocin inside the lipid bilayer, was also confirmed by MD simulations. Figure 7 illustrates the localization of the crocin molecule in the model DMPC membrane. One can see that hydrophilic groups of crocin (atoms 1 and 2 in Figure 7) are located near the surface of the membrane, but the hydrophobic chain (atom 3) penetrates deeper into the polar region of the lipid bilayer and is located near the phospholipid acyl chain but does not make contact with the terminal CH_3_-groups of the lipid. The lipid bilayer is centered at the center of the box.

**Figure 8:**
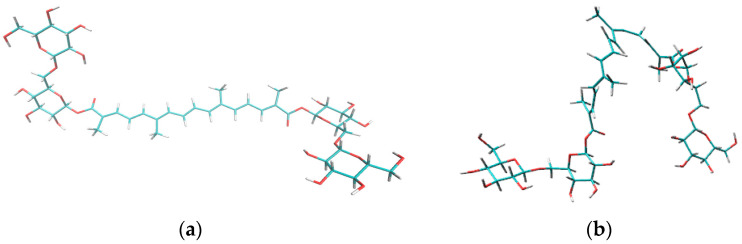
Configuration of crocin molecule: (**a**) initial configuration in water, (**b**) configuration in lipid bilayer obtained by MD simulation.

Based on the sNOESY experimental data and the MD calculations, we assume that the crocin molecule in the lipid bilayer has a curved shape, as earlier suggested by Khan and coauthors for crocin molecules incorporated into CTAB micelles [13]. Since in the lipid membrane, the crocin molecule changes its form to a U-shape (see Figure 8), the sugar fragments become not equivalent. Therefore, atoms 1 and 2, which stay in symmetric positions outside the membrane, show the three distribution peaks shown in Figure 7. The conclusions about the shape of the crocin molecule are made on the basis of observed NOE signals. When the crocin –CH= protons were saturated, NOE signals of the phospholipid CH_2_ protons were observed. However, we did not observe cross-peaks between the –CH= protons of crocin and the terminal CH_3_ protons of the phospholipid, which would be expected if the crocin molecule were in an unfolded form. We also confirmed the curved form of the crocin molecule by MD simulations (see Figure 8). Note, the U-shape is a characteristic form for bolaamphiphiles like the crocin molecule.

### 2.3. Peroxidation of Linoleic Acid Micelles in the Presence of Crocin

In the last decade, several studies have indicated that carotenoids may prevent or inhibit certain types of cancer, atherosclerosis, age-related muscular degeneration, and other diseases (see the last review as an example [46]). It was suggested that such activity of carotenoids is related to their ability to scavenge free radicals. A number of studies have indicated that carotenoids act as antioxidants in solution, micelles, and lipid membranes. Three mechanisms are usually discussed for the reaction of free radicals with carotenoids, namely radical addition to the carotenoid polyene chain, hydrogen abstraction from the carotenoid, and electron transfer. The primary products of these reactions were further reacted with other reactive species by means of various reaction mechanisms to give a variety of products [46].

**Figure 9:**
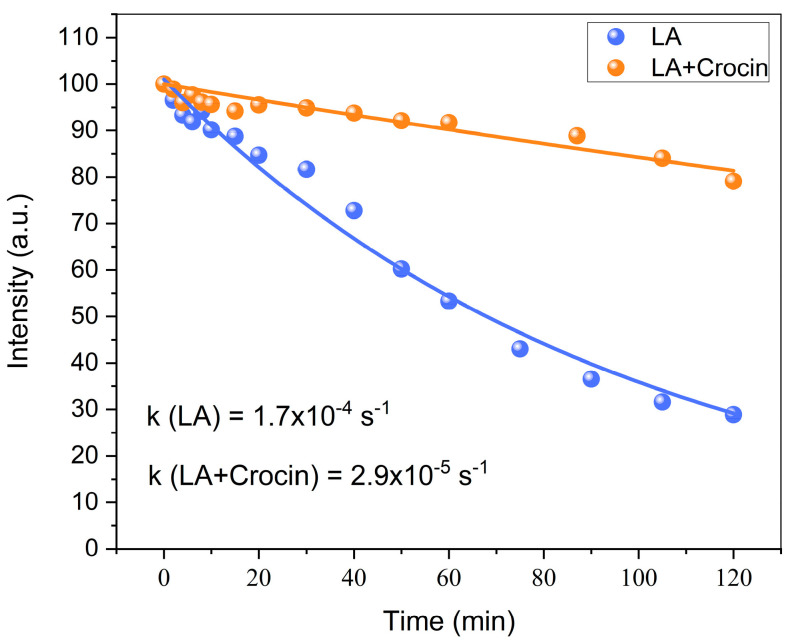
Kinetics of the linoleic acid oxidation detected by NMR signal at 2.7 ppm. Conditions: 1.0 mM crocin in PBS + 0.1 mM FeSO_4_ + 1 μL LA + 20 μL H_2_O_2_.

In the present work, the antioxidant activity of crocin was studied through the NMR method using LA micelle oxidation as a model process (Equations (1)–(4)). Lipid micelles formed by LA are widely used as models to simulate the properties of biological membranes [46]. Likewise, the polyunsaturated fatty acid LA has also been used in lipid peroxidation studies [47,48]. We studied the redox activity of crocin in an LA peroxidation reaction in the presence of metal ions through the changes in the NMR spectrum at different time delays after mixing. The changes in the LA proton signal intensity during the reaction were recorded to conduct a kinetic analysis of the redox process [49]. Specifically, we performed a kinetic analysis of the reaction process through the time-dependent change in the NMR signal intensity of the bisallyl protons (2.7 ppm, see Figure 5) of LA. Because the bisallyl group of LA loses a hydrogen atom in the initial stage of oxidation, and the reaction products (Equations (1)–(4)) have no NMR signal at 2.7 ppm, the initial stage of the reaction can be characterized. The intensity of the signal at 2.7 ppm decreases with time (Figure 9). The following Equations (1)–(4) represent the iron-induced lipid peroxidation process [50,51,52,53].

Fe^2+^ + H_2_O_2_ → Fe^3+^ + OH + OH^−^ (1)

LH + OH·→ L + H_2_O (2)

L + O_2_ → LOO·

LOO + LH → LOOH + L(3)

LOOH → LO·→ epoxides, hydroperoxides, aldehydes

L·+ L·→ L − L(4)

LOO + L → LOOL LOO + LOO → LOOL + O_2_

In addition, we studied the effect of crocin on lipid oxidation in the presence of vitamin C (ascorbate). The influence of ascorbate on this process is mediated by the reduction of Fe^3+^ to Fe^2+^ (5), which accelerates the formation of OH radicals (1):

AscH^−^ + Fe^3+^ → Asc•^−^ + Fe^2+^ + H^+^ (5)

From Figure 9 we can see that the signal intensity decays exponentially with time. Then, through data processing, we obtained the rate constant of the LA peroxidation reaction. In the absence and presence of crocin, the rate constants of the initial stage of LA peroxidation are 1.7 ± 1 × 10^−4^ s^−1^ and 2.9 ± 0.2 × 10^−5^ s^−1^, respectively, from which we can infer that crocin, as an antioxidant, can slow down the oxidation of lipids. Interestingly, when we measured the redox activity of Na_2_GA and the crocin/Na_2_GA mixture in the peroxidation reactions of LA (Table 1), we found that both Na_2_GA and the crocin/Na_2_GA complex decreased the oxidation rate with the same rate constants. This shows that Na_2_GA itself has an obvious antioxidant effect, as it was previously detected by EPR and CIDNP techniques.

**Figure 10:**
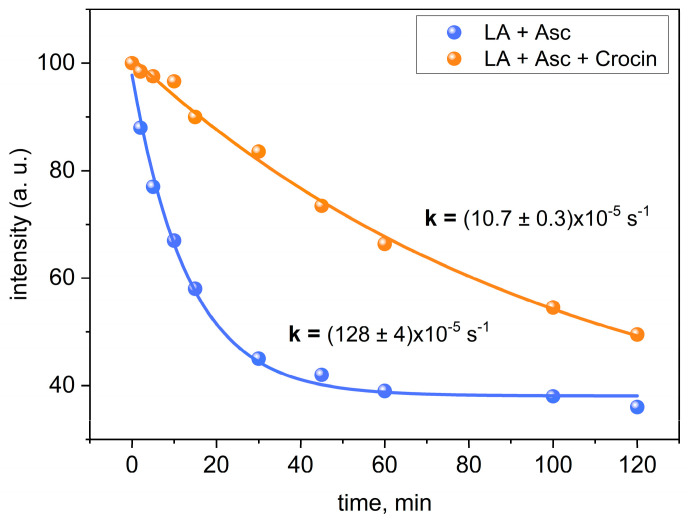
Ascorbate enhanced LA peroxidation in the presence and in the absence of crocin at 20 °C. Peroxidation studies were performed using 3.5 mM LA, 1 mM crocin, 0.1 mM FeSO_4_ (pH 7.4), 2.5 mM ascorbate, and 0.5 M H_2_O_2_. Graphs are based on the decay of the integral intensity of LA protons at 2.7 ppm.

The addition of ascorbic acid significantly increased the rate constant of LA peroxidation by a factor of 7.5 compared to similar conditions without ascorbic acid (see Table 1 and Figure 10). This observation may be due to the rapid conversion of Fe(III) to Fe(II) in the presence of ascorbic acid. This accelerates the formation of OH radicals via the Fenton reaction (1). In the presence of ascorbic acid and crocin at the same time, the rate constants of LA peroxidation were significantly lower than in the control group without the addition of ascorbic acid. The results of this experiment indicate that crocin is able to inhibit the pro-oxidant effect of ascorbic acid in metal-induced lipid peroxidation. 

## 3. Materials and Methods

### 3.1. Materials 

Crocin was purchased from Hangzhou Muhua Biotechnology Co., Ltd., Hangzhou, China. Glycyrrhizic acid (GA, purity of GA ~98.0%) and Na_2_GA (purity of Na_2_GA ~99.5%) were purchased from Shaanxi Panier Biotechnology Co., Ltd., Xi’an, China. Linoleic acid was purchased from Shanghai Aladdin Bio-Chem Technology Co., Ltd., Shanghai, China. The lipids 1,2-dimyristoyl-sn-glycero-3-phosphocholine (DMPC) and 1,2-dihexanoyl-sn-glycero-3-phosphocholine (DHPC) (both >99% purity) were purchased from Avanti Polar Lipids, Inc., Birmingham, AL, USA. Deuterated solvent (D_2_O, 99.8% D) was obtained from Sigma Aldrich (St. Louis, MO, USA) and was used as supplied. 

### 3.2. NMR and Selective NOESY Measurements

The ^1^H NMR and selective gradient NOESY (sNOESY) spectra were recorded on a Bruker AVHD-500 (500 MHz at ^1^H) NMR spectrometer (Bruker, Billerica, MA, USA) at 300 °K. The optimal mixing time, that is, the delay during which the magnetization transfer via cross-relaxation occurs in selective NOESY, was determined through a series of sNOESY experiments. The NMR spectra were processed using TopSpin 3.6.2 software. The T_2_ relaxation time was measured using a Carr–Purcell–Meiboom–Gill pulse sequence at 300 K. The spin–spin relaxation time T_2_ is closely related to the mobility of the molecule and is inversely proportional to the rotation correlation time. Therefore, by using T_2_ data, we can draw conclusions about the influence of the environment or the molecular state (free/bound).

### 3.3. Sample Preparation for Lipid Peroxidation Studies

Micelles of LA (3.5 mM LA) were used as the model for lipid peroxidation. Solutions of 0.5 M H_2_O_2_, 0.1 mM FeSO_4_ prepared in phosphate-buffered saline (PBS), and 2.5 mM ascorbic acid were used for the lipid peroxidation studies of crocin. All the experiments were conducted at the natural oxygen level.

### 3.4. Sample Preparation for NMR Study of Crocin Interaction with Bicells

Small isotropic DMPC/DHPC bicelles were used as a model of crocin interaction with cell membranes. The bicelles were formed from DMPC (1,2-dimyristoyl-sn-glycero-3-phosphocholine) and DHPC (1,2-diheptanoyl-sn-glycero-3-phosphocholine). Powdered lipids were dissolved in chloroform, the solvent was dried, and the resulting film was hydrated with PBS (pH = 7.4) dissolved in D_2_O. To accelerate the formation of bicelles, three freeze–thaw cycles were performed [54]. The DMPC:DHPC ratio was 1:2, with the total lipid concentration being 12 mM.

### 3.5. Molecular Dynamics Simulations

Molecular dynamics simulations were performed to understand the interactions of crocin with phospholipid-containing membranes using the GROMACS 2018.4 package and GROMOS54a7 force field. The topology of crocin was built using the Automated Topology Builder [55]. For lipid simulations, the model DMPC lipid was utilized [56]. The simple point charge model of water molecules was used.

The simulation was performed in the NPT ensemble with constant pressure (1 bar) and constant temperature T = 300 K, which were maintained by the semi-isotropic Parrinello–Rahman barostat [57] and Nose–Hoover thermostat [58]. The initial configuration of the system contained the bilayer consisting of 128 lipid molecules surrounded by water (~10,000 water molecules) and a crocin molecule located in water outside the bilayer. One production run of 500 ns duration was performed.

## 4. Conclusions

In the present study, using various NMR techniques (T_2_ relaxation and selective gradient NOESY), we have shown that crocin can form mixed micelles with other micelle-forming amphiphilic molecules, namely saponins (glycyrrhizic acid) and lipids (linoleic acid). Note that both saponins and lipids are capable of forming nanoscale associates used as drug delivery systems. In addition, it was demonstrated that the crocin molecule was not able to penetrate the center of the membrane but was located mainly near the membrane surface. Based on the sNOESY experimental data and the MD simulation, we assume that the crocin molecule in the lipid bilayer, as well as in the mixed micelles with LA and GA, has a curved shape, as previously suggested by Khan and coauthors for crocin molecules incorporated into CTAB micelles [13]. In the case of the lipid bilayer, the hydrophilic sugar groups of crocin were located near the surface of the membrane, but the hydrophobic chain penetrated deeper into the polar region of the lipid bilayer and was located near the phospholipid acyl chain but still did not make contact with the terminal CH_3_-groups of the lipid.

Based on our preliminary experience, we can expect that the incorporation of crocin into these mixed micelles can significantly increase crocin penetration through lipid membranes as well as enhance its stability [59]. We plan to elucidate these aspects in the next study. Some authors suggested that crocin micelles could be a promising compound for studying nonlinear photodynamic therapy [60]. From this point of view, the location of crocin in the cell membrane and its ability to penetrate through the lipid bilayer are also very important.

Additionally, the NMR technique has been applied to demonstrate the antioxidant activity of crocin and glycyrrhizic acid using the model reaction of iron ion-induced lipid peroxidation. We have studied the influence of crocin, glycyrrhizic acid, and ascorbic acid on lipid peroxidation in a model system—the micelles of linoleic acid that mimic the cell membrane. The direct calculation of the rate constant of the initiation stage of lipid peroxidation was performed using the original approach based on the measurement of the decay rate of the NMR signal of the bis-allylic proton of linoleic acid [61].

The results of this study may be useful for expanding the field of application of crocin in medicine and in the food industry.

## Figures and Tables

**Table 1:** Rate constants of the reactions of linoleic acid (LA) peroxidation induced by iron ions in the absence and in the presence of crocin, Na_2_GA and ascorbate.

System	Rate Constant, in 10^−5^ s^−1^
LA	17 ± 1
LA + 1 mM crocin	2.9 ± 0.2
LA + 1 mM Na_2_GA	1.8 ± 0.2
LA + 0.1 mM crocin + 1 mM Na_2_GA	1.8 ± 0.2
LA + 2.5 mM ascorbate	128 ± 4
LA + 1 mM crocin + ascorbate	10.7 ± 0.3

## Data Availability

The data are contained within the article.
